# The Release of Non‐Native Gamebirds Is Associated With Amplified Zoonotic Disease Risk

**DOI:** 10.1111/ele.70115

**Published:** 2025-04-21

**Authors:** Emile Michels, Kayleigh Hansford, Sarah E. Perkins, Robbie A. McDonald, Jolyon M. Medlock, Barbara Tschirren

**Affiliations:** ^1^ Centre for Ecology and Conservation University of Exeter Cornwall UK; ^2^ Medical Entomology and Zoonoses Ecology Group UK Health Security Agency Salisbury UK; ^3^ School of Biosciences Cardiff University Cardiff UK; ^4^ University of Exeter, Environment and Sustainability Institute University of Exeter Cornwall UK

**Keywords:** *Borrelia burgdorferi* sensu lato, environmental change, invasive species, one health, spillover, zoonosis

## Abstract

Spillback—where non‐native species increase native pathogen prevalence—is potentially an important mechanism by which non‐natives contribute to zoonotic disease emergence. However, spillback has not yet been directly demonstrated because it is difficult to disentangle from confounding factors which correlate with non‐native species abundance and native pathogen prevalence. Here, we capitalise on replicated, quasi‐experimental releases of non‐native pheasants ( *Phasianus colchicus* ) to compare vector abundance and native pathogen prevalence between sites with similar local conditions but different non‐native densities. Prevalence of *Borrelia* spp. (the causative agent of Lyme disease) in questing ticks was almost 2.5x higher in woods where pheasants are released compared to control woods, with a particularly strong effect on *Borrelia garinii,* a bird specialist genospecies. Furthermore, adult (but not nymphal) ticks tended to be more abundant at pheasant‐release woods. This work provides evidence that non‐native species can impact zoonotic pathogen prevalence via spillback in ecologically relevant contexts.

## Graphical abstract

Spillback is potentially an important mechanism by which non‐natives contribute to zoonotic disease emergence. We capitalise on quasi‐experimental releases of non‐native pheasants ( *Phasianus colchicus* ) to compare native pathogen prevalence between sites with similar local conditions but different non‐native densities. Prevalence of *Borrelia* sp. (the causative agent of Lyme disease) in questing ticks was almost 2.5× higher in woods where pheasants are released, with a particularly strong effect on *Borrelia garinii* .

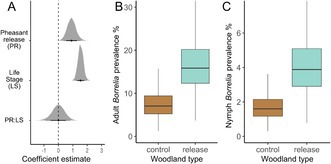

## 1 Introduction

Rapidly accelerating biodiversity loss and the expansion of international trade have left ecosystems susceptible to the introduction, establishment and spread of non‐native species (Kennedy et al. 2002; Sardain et al. 2019; Stachowicz et al. 2002). Evidence is mounting that these processes play a key role in the emergence and re‐emergence of zoonotic diseases (Roy et al. 2023; Zhang et al. 2022), by providing an opportunity for new parasite–host interactions to establish or by changing the dynamics of existing disease transmission pathways (Young et al. 2017). Non‐natives can introduce pathogens from their native range to novel hosts (‘spillover’; Power and Mitchell 2004; Nanetti et al. 2021), amplify native pathogens (‘spillback’; Kelly et al. 2009), or alter disease dynamics by changing the composition of native host/vector communities (Burkett‐Cadena et al. 2021; Hoyer et al. 2017).

Spillback is likely the most common of these mechanisms, yet it has received less attention than spillover and is generally less well understood (Kelly et al. 2009). One potential reason is that spillback is difficult to conclusively demonstrate. Observations of non‐native species hosting native pathogens are often used as evidence of spillback (Bezerra‐Santos et al. 2023). However, to affect disease dynamics via spillback, non‐native species not only need to be susceptible to infection by a native pathogen but must also effectively transmit the pathogen (Downs et al. 2019). Furthermore, given the diversity of effects which non‐natives can exert on native host community composition (Hoyer et al. 2017), the overall effect of non‐natives on a native pathogen's prevalence is difficult to predict (Swei et al. 2012). To demonstrate spillback, measuring the prevalence of a zoonotic pathogen in native hosts/vectors is imperative and, in the case of vector‐borne pathogens, measuring and accounting for changes in vector abundance is also crucial.

Demonstrating spillback is also complicated because the effects of non‐native species on disease dynamics are often confounded by correlated factors (Carlson et al. 2022; Halliday et al. 2020; Young et al. 2017; Zhang et al. 2022). For instance, anthropogenic disturbance is associated with both high non‐native abundance (MacDougall et al. 2014) *and* increased disease prevalence independent of species introductions, for example, by changing native host community composition (Ostfeld 2009), eliciting stress in native hosts (Narayan 2019) or changing native host densities (Mbora and McPeek 2009). Studying non‐native species deliberately introduced for recreational or commercial activities, such as hunting or fishing, provides an opportunity to overcome this complication. These deliberate introductions are common: > 20% of non‐native species are thought to have been translocated and to be restocked for recreational activities (Carpio et al. 2017; Jeschke and Strayer 2006). Compared to accidentally introduced species, these deliberately introduced non‐natives are less likely to spread (Jeschke and Strayer 2006), and thus tend to have clustered distributions in a landscape. As such, pathogen prevalence in native hosts/vectors can be compared between locations of high and low non‐native density, but with similar local conditions (i.e., climate, anthropogenic disturbance and land‐use). Furthermore, in the context of invasion biology, deliberately introduced non‐natives also act as a more ecologically relevant model than fully domestic non‐native species, which also often interact with wildlife disease transmission cycles (Bouwmeester et al. 2021; Ayala et al. 2020).

In the UK, around 47 million common pheasants ( *Phasianus colchicus* Linnaeus 1758) are released each year for recreational shooting (Aebischer 2019). The biomass of pheasants in late summer is estimated to be equivalent to that of all native UK breeding birds combined (Blackburn and Gaston 2021) but the ecological consequences of these releases are still poorly understood (Madden et al. 2023). One potential consequence of pheasant release is the amplification of zoonotic pathogens, in particular *Borrelia burgdorferi* sensu lato (Johnson et al. 1984; Kurtenbach, Carey, et al. 1998; Kurtenbach, Peacey, et al. 1998). *Borrelia burgdorferi* s.l. is the causative agent of Lyme disease, the most prevalent vector‐borne zoonotic disease in the northern hemisphere (Lindgren and Jaenson 2006). Pheasants can harbour hundreds of *Ixodes ricinus* (Linnaeus 1758) ticks (Hoodless et al. 2002), the vector of *B. burgdorferi* s.l., and experimental trials in captivity have demonstrated that pheasants can contract and re‐transmit *B. burgdorferi* s.l. to and from *I. ricinus* (Craine et al. 1997; Kurtenbach, Carey, et al. 1998; Kurtenbach, Peacey, et al. 1998). However, the impact of pheasant release on tick abundance and *Borrelia* sp. prevalence in ticks, in ecologically relevant contexts, has not yet been quantified.

Here we test whether the release of these non‐native birds affects the amplification of zoonotic pathogens. Specifically, we compare the abundance of ticks and the prevalence of *B. burgdorferi* s.l. in ticks between woodlands (hereafter referred to as ‘woods’) where pheasants are released, and paired woods where no pheasants are released. To better understand the relative importance of direct spillback effects vs. indirect effects (e.g., changes in host community composition), we also determine which *Borrelia* genospecies are most affected by pheasant‐release. We predict higher *B. burgdorferi* s.l. prevalence in ticks from pheasant‐release woods and for the bird‐specialist *Borrelia garinii* (Baranton et al. 1992) (Hanincová et al. 2003) to be most strongly amplified.

## 2 Materials and Methods

### 2.1 Pheasant‐Release for Recreational Shooting

In the United Kingdom, common pheasants ( *P. colchicus* ) are released each year for recreational shooting (Aebischer 2019). Organisations that release pheasants are referred to as ‘shoots’. In summer, shoots release juvenile pheasants into ‘release pens’—fenced enclosures within woods. The number of pheasants released varies greatly, ranging from a few hundred to over 100,000 birds per shoot. Release pens feature small gates through which pheasants can exit into the surrounding woodland and re‐enter freely from the moment of their release. Shoots may use one pen in a single wood, multiple pens within a single wood or multiple pens across separate woods. Because pens are costly to construct, they are reused annually for up to 20 years before being re‐constructed or relocated (Draycott et al. 2006; Sage et al. 2005).

In release pens, pheasants are provisioned with food, water and shelter. Provisioning typically continues until the end of the shooting season on the 1st February. Some shoots continue to provision pheasants beyond the shooting season's end, with the aim of establishing feral populations, whereas others proactively cull remaining pheasants in an attempt to reduce inter‐annual disease transmission between pheasant cohorts. In either scenario, pheasant mortality is high. It is estimated that only 9% of pheasants are still alive in spring compared to their peak abundance in late summer (Blackburn and Gaston 2021).

### 2.2 Sampling Sites

**FIGURE 1:**
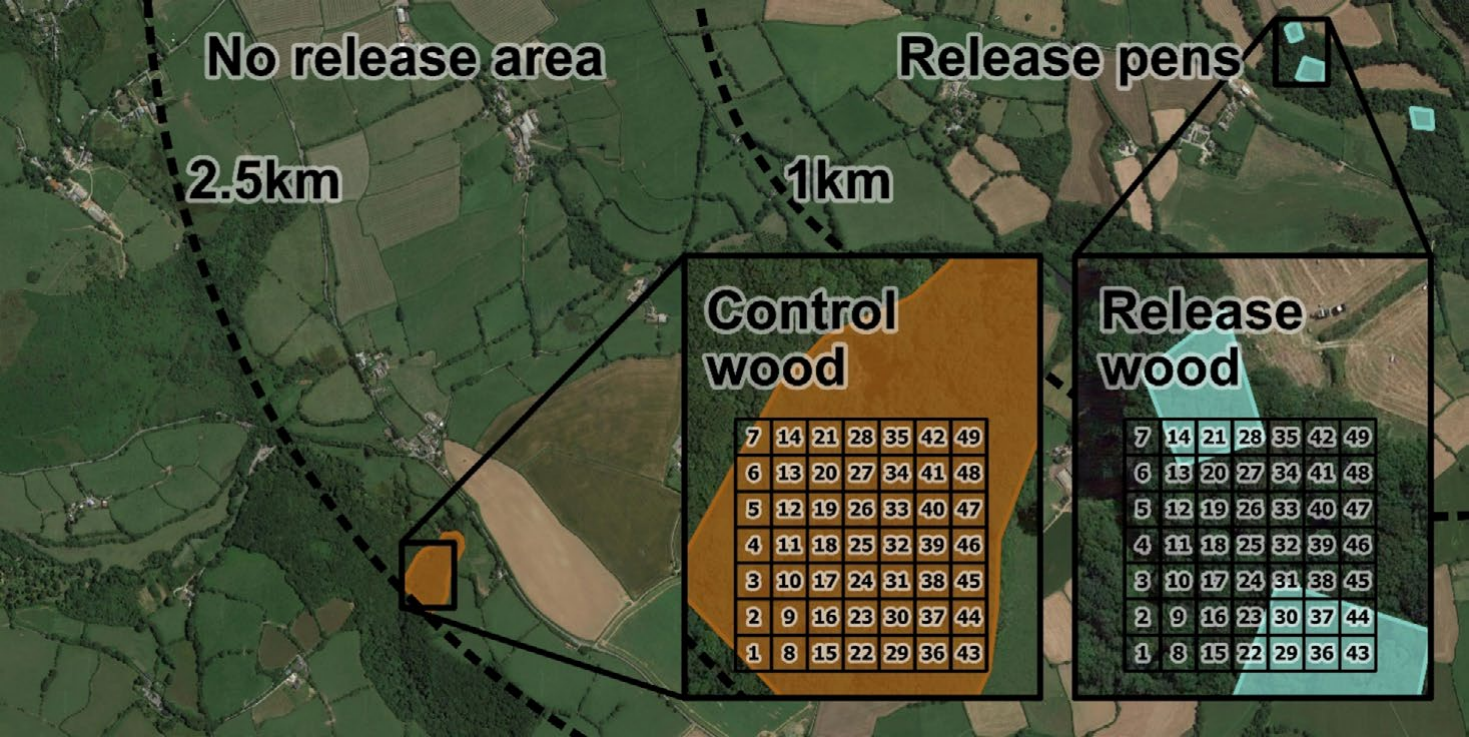
An aerial map of a representative pheasant shoot. Small blue compartments show the area outline of release pens. 1 km and 2.5 km perimeters are drawn around each release pen. Between the two perimeters is a ‘no pheasant release’ area, within which a control wood is highlighted in brown. Inset maps show zoomed in views of a release and a control wood with a 7 × 7‐cell sampling grid overlaid.

Twenty‐five shoots from the southwest of England were included in this study, capturing the range of pheasant‐release magnitude (median: 5000; IQR: 2000—20,500; range: 300–105,000 released birds) that exists across shoots. Each shoot was visited during a period of high tick activity (10 May—16 July 2022). At each shoot, we aimed to collect ticks from two woods in which pheasants had been released during the summer preceding tick collection (hereafter referred to as ‘release woods’) and two woods where no pheasants were released (hereafter referred to as ‘control woods’). Control woods were between 1 and 2.5 km from the nearest known release pen, a distance outside of the typical post‐release dispersal range of pheasants (Turner 2008; Figure 1). Control and release woods could either make up four parts of one contiguous woodland habitat or be isolated woodlands separated by fields or roads. We verified the suitability of control woods by confirming the absence of any visible signs of pheasant‐release using Google maps (i.e., fences or nearby cover crops; Madden and Sage 2020), consulting with local shoot managers to confirm there was no known history of pheasant‐release, and checking for visible signs of past or present pheasant‐release when woods were visited for tick sampling (i.e., supplementary feeders, release pen fences). At seven of the shoots only a single control wood was sampled that met these criteria. At four of the shoots, only a single release wood was sampled because all pheasants were released from a single pen. In total, ticks were collected from 89 woods (46 release woods and 43 control woods).

### 2.3 Tick Sampling

To quantify *I. ricinus* abundance in control and release woods, we dragged a 1 m^2^ white cotton sheet along 5 m transects over understory vegetation (i.e., covering a total area of 5 m^2^). For each transect (hereafter referred to as a ‘drag’), we collected all nymphal and adult ticks attached to the cloth and stored them in 97% ethanol. We completed 10 structured drags in each wood. The location of each drag was randomly selected from a 7 × 7 grid of 10 m^2^ cells created using QGIS (QGIS Association, n.d.) (Figure 1). Only one drag was completed in each cell. To improve the accuracy of our *Borrelia* sp. prevalence estimates, in woods where < 20 ticks were collected during the initial 10 structured drags (*n* = 59), we continued with *ad hoc* dragging until at least 20 ticks were collected. Tick abundance estimates were derived exclusively using data from the 10 initial structured drags. We did not collect ticks in their first life stage (larva), because *B. burgdorferi* s.l. is very rarely transmitted from adult females to offspring, and larvae thus play a minor role in the transmission of Lyme disease (Richter et al. 2012; Rollend et al. 2013). Using a morphological key (Hillyard 1996), we identified all ticks to species level and determined their life stage.

### 2.4 Ecological Variables

To allow us to control for factors that can influence tick abundance and *Borrelia* spp. prevalence independent of pheasant‐release, we recorded several ecological variables for each drag. We recorded the date upon which each tick was collected. We measured ambient temperature before each drag (Kahl and Gray 2023; Macleod 1936). Because of its effect on microclimate and consequently, tick activity (Macleod 1936), we characterised understory vegetation by measuring vegetation height at the midpoint of each drag using a wooden dowel marked at 10 cm intervals from 0 to 100 cm (Nelson et al. 2015) and by visually estimating the percentage of bare ground over the area dragged. We recorded whether the nearest tree was a conifer or broadleaf species and noted if the drag was conducted at the edge (≤ 2 m from non‐woodland habitat) or interior of the woodland (> 3 m from non‐woodland habitat) (Hansford et al. 2017, 2022). No drags were conducted between 2 and 3 m from the edge of woods. Finally, using QGIS (QGIS Association, n.d.) we quantified the total continuous area (m^2^) of the woods we sampled (i.e., unbroken by fields, large roads or rivers).

### 2.5 *Borrelia* spp. Screening and Genospecies Differentiation

We extracted DNA from ticks using previously published protocols (Hansford et al. 2015). We then used a pan‐*Borrelia* qPCR assay targeting the 16 s rRNA gene to screen ticks for all pathogenic *Borrelia* genospecies (Medlock et al. 2022; Parola et al. 2011). To reduce screening costs, we pooled ticks before DNA extraction and *Borrelia* sp. screening. Each pool contained two ticks of the same life stage, collected from the same wood and at the same distance from the woodland edge. Single ticks left over after pooling were extracted and screened individually. We considered all samples with CT values < 39 as *Borrelia* spp. positive. The probability of individual ticks carrying *Borrelia* spp. was modelled statistically using pooled and individual results (Methods S1). To quantify *Borrelia* spp. detectability and cross‐contamination during DNA extraction or qPCR, each qPCR plate included one positive control (purified *Borrelia* sp. DNA), a negative qPCR control (distilled water), and 12 extraction controls (ammonium hydroxide only). To determine *Borrelia* genospecies, positive samples were sequenced at the 5S‐23S rRNA intergenic spacer region as described by Hansford et al. (2023).

### 2.6 Statistical Analysis

Statistical analyses were completed in a Bayesian framework. For each variable, we report the mean coefficient estimate from posterior distributions along with the 95% highest posterior density intervals (HPDIs).

Separate models were constructed to test the effects of pheasant‐release on tick abundance, *Borrelia* spp. prevalence in ticks, and genospecies‐specific *Borrelia* prevalence in ticks. To improve our ability to detect these effects, and to disentangle direct effects of pheasant‐release from those mediated by ecological variation across woods, we included ecological variables as covariates. To identify which ecological variables to include, we created models which featured only ecological variables as predictors, then excluded those with 79% HPDIs spanning zero, leaving what we here refer to as the ‘ecological null model’ (details in Results S1, Figure S1).

### 2.7 Tick Abundance Model

Tick abundance was modelled as a multivariate negative binomial response, with nymph and adult abundances modelled as distinct observations from a single drag. The ecological null model included woodland size (area in m^2^), vegetation depth, nearest tree species (conifer vs. broadleaf), and percentage bare ground (Figures S1A,B). We then added pheasant‐release (release vs. control woods) as a binary predictor to determine differences in adult and nymph abundances between control and pheasant‐release woods. We used posterior contrasts (i.e., the difference in posterior distributions) to determine if the effect of pheasant release differs between the two life stages. The model included random effects to account for non‐independence *within* woods (*n* = 89) and shoots (*n* = 25), and a Gaussian process to account for spatial autocorrelation *among* shoots.

### 2.8 *Borrelia* sp. Prevalence Model

Tick *Borrelia* sp. prevalence (i.e., non‐genospecies‐specific prevalence) was modelled as a binomial response at the level of individual ticks. The ecological null model included distance from the woodland edge (Figure S1C). We then added tick life stage (nymph vs. adult) and pheasant release (release wood vs. control wood) as binary predictors and tested for an interaction between the two. We used a custom binomial distribution to account for two potential sources of *Borrelia* spp. misclassification (McElreath 2020, Chapter 17). These are: false positives occurring due to *Borrelia* spp. negative and positive ticks being pooled together, and false positives due to a 0.05% chance of cross‐contamination during DNA extraction/qPCR (Methods S1). This approach allowed us to derive more accurate estimates of effect sizes than would be possible using minimum/maximum‐possible prevalence (Fracasso et al. 2023). The model also included random effects to account for non‐independence within woods (*n* = 89) and shoots (*n* = 25), and a Gaussian process to account for spatial autocorrelation among shoots.

### 2.9 Genospecies‐Specific *Borrelia* Prevalence Model

To determine if different *Borrelia* genospecies respond differently to pheasant‐release, we used a multinomial model with five outcomes: *B. garinii‐*infected, *B. afzelii‐*infected (Canica et al. 1993), *B. valaisiana‐*infected (Wang et al. 1997), other/unresolved, and non‐infected. We could not account for misclassification in this model; instead, we used maximal possible prevalence as a response variable, with an offset for tick pool size. Maximal possible prevalence was approximate as the genospecies of 25 samples could not be resolved. We included the predictors tick life stage (nymph vs. adult) and pheasant‐release (control woods vs. release woods), and random effects of wood (*n* = 89) and shoot (*n* = 25). To ensure unbiased posterior exploration, this model did not feature a Gaussian process accounting for spatial autocorrelation among shoots.

### 2.10 Sensitivity Analyses and Release Magnitude Effects

We completed sensitivity analyses to determine if the estimated effect of pheasant release on tick abundance and *Borrelia* spp. prevalence depends upon the inclusion of specific ecological variables (Results S2, Figure S2). Furthermore, we performed additional analyses to explore the impact of release magnitude (i.e., the number of pheasants released at a shoot) on tick abundance and *Borrelia* sp. prevalence (Results S3; Figure S3).

All analyses were completed in R version 4.1.0 (R Core Team 2023), using the packages *‘rethinking’* (McElreath 2020) and ‘brms’ (Bürkner 2021)—compilers for the STAN modelling platform (Stan Development Team 2023). All models were implemented using 4000 samples from four chains. Model diagnostics, including trace plots and R‐hat values (< 1.01), showed no issues with convergence or biased posterior exploration.

## 3 Results

### 3.1 Tick Abundance

**FIGURE 2:**
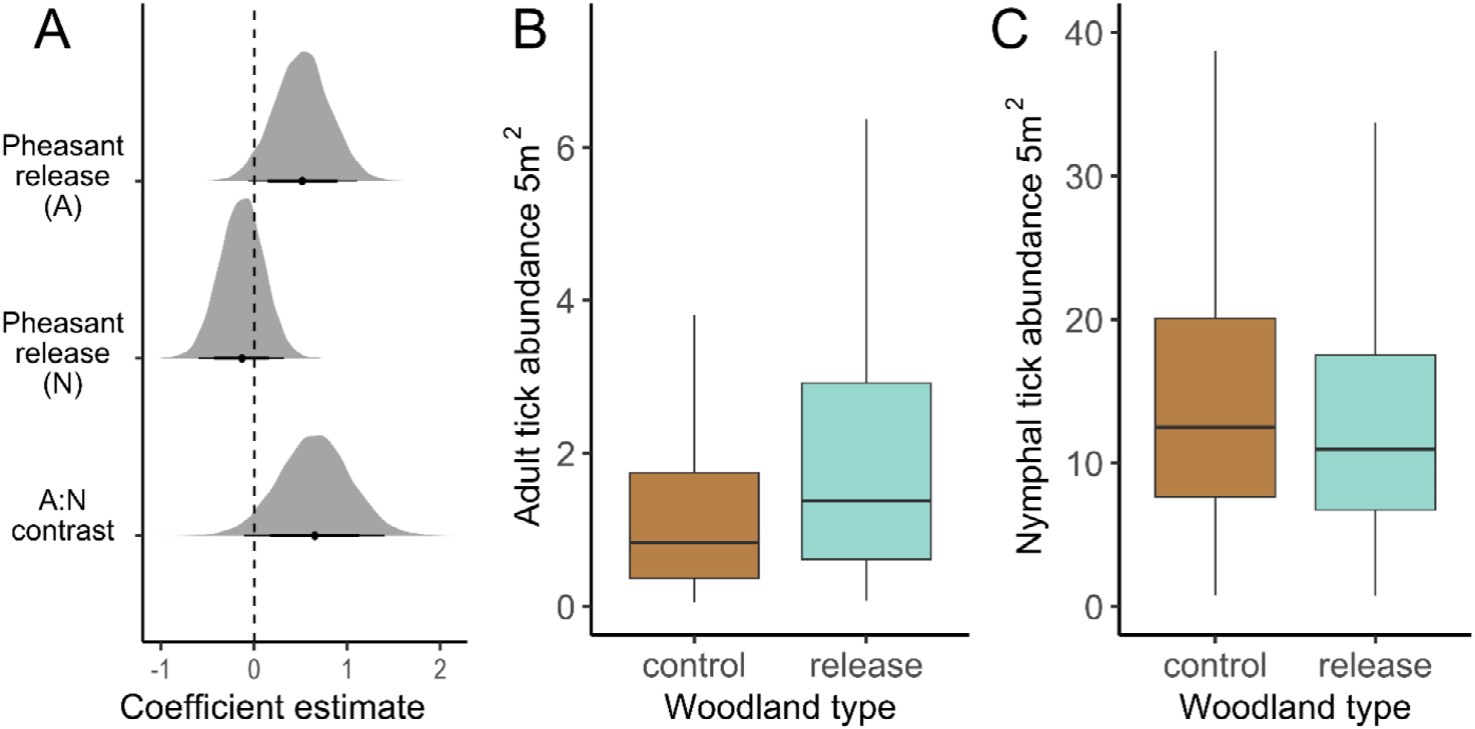
(A) Posterior distributions outlining the effect of pheasant‐release on *Ixodes ricinus* abundance, derived by comparing *I. ricinus* abundances between woods where pheasants are released and control woods where no pheasants are released. From top to bottom, the effect of pheasant‐release on adult tick abundance, the effect of pheasant‐release on nymphal tick abundance and, the difference between the effect of pheasant‐release on nymphs and adults. Black points correspond to the means of posterior distributions and horizontal lines represent 79% and 95% highest probability density intervals. For the upper and middle rows, positive values indicate higher abundance at woods where pheasants are released. (B, C) Predicted abundance *of I. ricinus* adults (B) and nymphs (C) collected at woods where no pheasants are released (brown boxes) and woods where pheasants are released (blue boxes). Central horizontal lines represent posterior distribution means, boxes and vertical lines encompass all predictions within one and two standard deviations of the mean, respectively. Predictions are conditional on random effects and ecological variables being held at mean values (for more information see Results S1).

During structured drags, 693 nymphs and 54 adults were collected from control woods (*n* = 43) compared to 671 nymphs and 110 adults from release woods (*n* = 46). All ticks were identified as *I. ricinus* . Adult tick abundance tended to be higher at pheasant‐release woods compared to control woods (mean effect: 0.52, 95% HPDI: −0.07 to 1.11 [log scale]; Figure 2A), whereas no effect on nymph abundance was observed (mean effect: −0.13, 95% HPDI: −0.60 to 0.32 [log scale]; Figure 2A). The contrast between these effects does not strongly suggest that the effect of pheasant‐release differs between the two life stages (mean effect: 0.65, 95% HPDI: −0.13 to 1.37 [log scale]; Figure 2A). These results were not contingent on the inclusion of ecological variables (Results S2; Figure S2) and did not change when a pheasant‐release × release magnitude interaction was added to the model (Results S3). Overviews of the posterior estimates for all parameters are shown in Figure S4A,B.

### 3.2 *Borrelia* Spp. Prevalence

**FIGURE 3:**
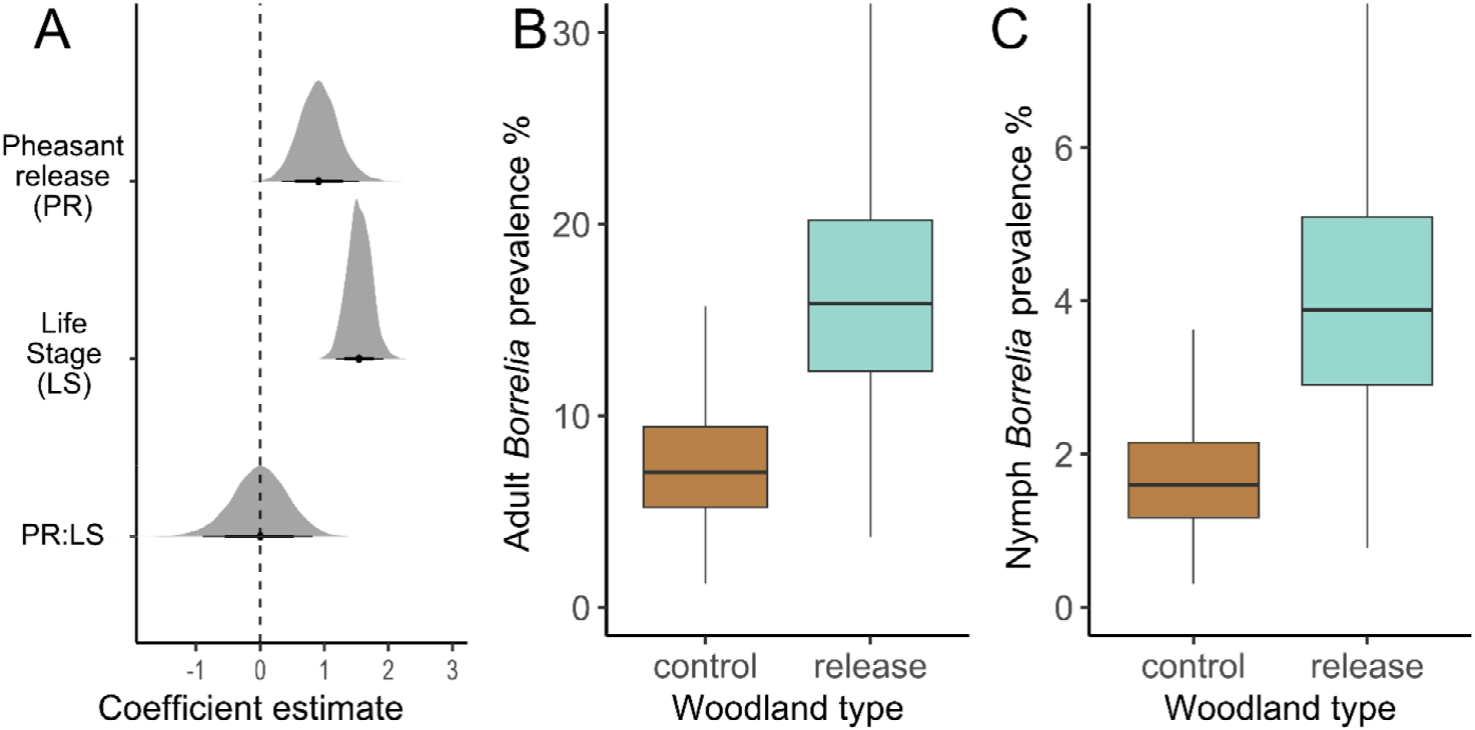
(A) Posterior distributions (from top to bottom) for the average difference in *Borrelia* sp. prevalence in *Ixodes ricinus* ticks between woods where pheasants are or are not released (pheasant‐release, PR). Positive values indicate higher prevalence in release woods. The difference in *Borrelia* sp. prevalence between nymphs and adults (LS), positive values indicate higher prevalence in adults. The difference between the effect of pheasant‐release (PR) on *Borrelia* sp. prevalence in nymphs and adults (LS). Black points correspond to the means of posterior distributions and horizontal lines represent 79% and 95% HPDI intervals. (B, C) Predicted *Borrelia* sp. prevalence in *I. ricinus* adults (B) and nymphs (C) collected at woods where no pheasants are released (brown boxes) and woods where pheasants are released (blue boxes). Central horizontal lines represent posterior distribution means, boxes and vertical lines encompass all predictions within one and two standard deviations of the mean, respectively. Predictions are conditional on random effects and ecological variables being held at mean values (for more information see Results S1). The predicted increase in the relative prevalence of *Borrelia* sp. infection, associated with pheasant release, is the same for adults and nymphs (2.45 times greater), but the absolute increase in prevalence differs between the two life stages.

A total of 2493 (343 adults and 2150 nymphs) *I. ricinus* ticks were screened for *Borrelia* spp. We found strong evidence that nymph and adult ticks collected from pheasant‐release woods were more likely to be infected with *Borrelia* spp. (mean effect: 0.92, 95% HPDI: 0.33–1.5 [logit scale]; Figure 3A). *Borrelia* spp. prevalence in ticks collected from pheasant‐release woods was 2.45 times higher than for ticks collected from control woods (mean percentage increase: 144.58%, 95% HPDI: 19.18%–291.6%; Figure 3B,C). We also found strong evidence that *Borrelia* spp. prevalence was higher in adults than in nymphs (mean effect: 1.55, 95% HPDI: 1.15–1.88 (logit scale); Figure 3A) but no evidence that pheasant‐release affected *Borrelia* spp. prevalence in adults and nymphs differently (mean effect: −0.03, 95% HPDI: −0.87 to 0.79 (logit scale); Figure 3A). These results were not contingent on the inclusion of ecological variables in the model (Results S2; Figure S2) and did not change when a pheasant‐release × release magnitude interaction was added (Results S3). An overview of the posterior estimates for all parameters is shown in Figure S4C.

### 3.3 Genospecies‐Specific *Borrelia* Prevalence

**FIGURE 4:**
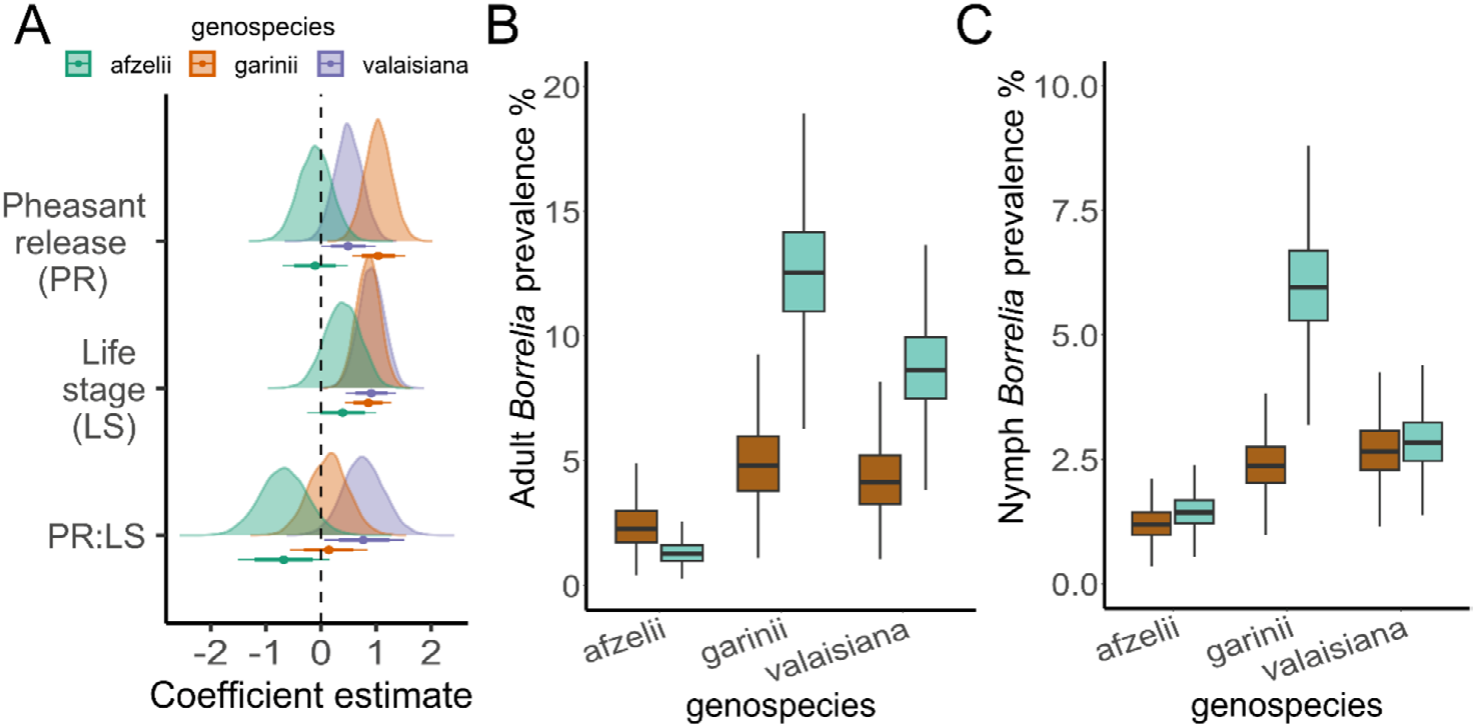
(A) Posterior distributions (from top to bottom) for the difference in *Borrelia* prevalence in *Ixodes ricinus* ticks between woods where pheasants are or are not released (PR) for the three most common genospecies in our samples (*Borrelia garinii, B. afzelii, B. valaisiana*), positive values indicate higher prevalence at pheasant‐release woods. The difference in *Borrelia* prevalence between nymphs and adults (LS), positive values indicate higher prevalence in adults. The difference between the effect of pheasant‐release on *Borrelia* prevalence in nymphs and adults (PR:LS), positive and negative values indicate greater amplification in adults and nymphs, respectively. Coloured points correspond to the means of posterior distributions and horizontal lines represent 79% and 95% highest probability density intervals. Summaries of posterior distributions and pairwise contrasts are presented in Tables S1 and S2. (B, C) Predicted maximum possible prevalence, for the three most common *Borrelia* genospecies in our sample (*B. garinii, B. afzelii, B. valaisiana*) in *I. ricinus* adults (B) and nymphs (C) collected at woods where no pheasants are released (brown boxes) and woods where pheasants are released (blue boxes). Central horizontal lines represent posterior distribution means, whilst boxes and vertical lines encompass all predictions within one and two standard deviations of the mean, respectively. Predictions are conditional on random effects and ecological variables being held at mean values (for more information see Results S1). The genospecies of 25 samples could not be resolved, due to co‐infection or low DNA quality, thus maximum possible prevalence values are approximate.

The *Borrelia* genospecies of 167/192 positive samples were successfully determined. The three most common genospecies in our sample were *B. garinii* (51% of infections), *B. valaisiana* (33% of infections) and *B. afzelii* (13% of infections). *B. garinii* (mean effect: 1.04, 95% HPDI: 0.57–1.51 [logit scale]; Figure 4A–C), and, to a lesser extent, *B. valaisiana* (mean effect: 0.5, 95% HPDI: 0–1 [logit scale]; Figure 4A–C) were amplified at release sites compared to control sites. There was no evidence that the prevalence of *B. afzelii* was affected by pheasant release (mean effect: −0.10, 95% HPDI: −0.68 to 0.49 [logit scale]; Figure 4A–C). In *B. valaisiana* , we found strong evidence for an interaction between pheasant release and tick life stage (mean effect: 0.77, 95% HPDI: 0.06–1.48 [logit scale]; Figure 4A), whereby *B. valaisiana* is amplified in adults but not in nymphs in release woods. Such interactions were not observed for *B. afzelii* (mean effect: −0.67, 95% HPDI: −1.5 to 0.12 [logit scale]; Figure 4A), or *B. garinii* , which was amplified in both life stages (mean effect: 0.14, 95% HPDI: −0.54 to 0.84 [logit scale]; Figure 4A). Summaries of all posterior distributions and contrasts are presented in Tables S1 and S2.

## 4 Discussion

Spillback is often proposed as an important mechanism through which non‐native species can influence the emergence of zoonotic and wildlife diseases. However, demonstrating spillback in ecologically relevant contexts is challenging. Here we show that the prevalence of *Borrelia* spp. in ticks, the causative agent of Lyme disease, is increased in woods where pheasants are released compared to woods where no pheasants are released. This effect was driven primarily by the amplification of *B. garinii*, a bird specialist genospecies which causes neuroborreliosis in humans (Balmelli and Piffaretti 1995; van Dam et al. 1993), and to a lesser extent by the amplification of *B. valaisiana* . We also show that there was a trend for an increase in adult (but not nymphal) tick abundance at pheasant‐release woods. These results suggest that the release of non‐native pheasants can amplify zoonotic disease risk via spillback.

Numerous previous studies have provided evidence for the *potential* of non‐native species to amplify zoonotic pathogens (Craine et al. 1995, 1997). Examples include the tapeworm *Echinococcus multilocularis* (Leuckart 1863), the causative agent of Alveolar Echinococcosis, found in non‐native rodents (Umhang et al. 2013), the human‐pathogenic roundworm *Baylisascaris procyonis* (Stefanski and Zarnowski 1951) found in non‐native raccoons ( *Procyon lotor* Linnaeus 1758) (Peter et al. 2023) or *Borrelia* spp. found in non‐native grey squirrels ( *Sciurus carolinensis* Gmelin 1788) (Craine et al. 1995) and Siberian chipmunk (*Eutamias sibiricus* Laxmann 1769) (Marsot et al. 2013). Indeed, analyses suggest that 36% of all non‐native species host at least one zoonotic pathogen (Zhang et al. 2022). However, to demonstrate pathogen amplification by non‐native species, it is imperative to quantify changes in pathogen prevalence in native vectors or hosts.

Recent studies have advanced in this direction. For example, a higher prevalence of Everglades virus (EVEV) has been observed in mosquito vectors since Burmese python ( *Python bivittatus* Kuhl 1820) have invaded the Florida Everglades. This effect is likely due to selective predation by Burmese pythons, causing a shift in native host community composition in favour of highly competent rodent hosts (Hoyer et al. 2017; Burkett‐Cadena et al. 2021). However, to our knowledge, our study is the first to quantify the impact of a non‐native species on zoonotic pathogen amplification in a quasi‐experimental setting across multiple introduction and control sites. Because of the limited dispersal range of pheasants (Turner 2008), shoots can be considered replicated, localised introduction events. This spatial replication adds to the robustness of our findings in comparison to studies where multiple samples may come from a single introduction event. The quasi‐experimental nature of gamebird releases and the paired release‐control design of our study also allows us to disentangle the effects of non‐natives per se from other ecological processes which may be correlated with zoonotic pathogen prevalence and facilitate the introduction and establishment of non‐natives (Young et al. 2017; Zhang et al. 2022), for example, land‐use change (Faust et al. 2018; Gottdenker et al. 2014), climate change (Carlson et al. 2022) and biodiversity loss (Halliday et al. 2020).

Multiple non‐mutually exclusive mechanisms may cause *Borrelia* sp. amplification at pheasant‐release woods. Through biological interactions such as predating on invertebrates (Neumann et al. 2015), acting as a food source for meso‐predators (Pringle et al. 2019), or altering woodland vegetation structure (Sage et al. 2009), pheasants alter the ecosystems to which they are introduced (Madden et al. 2023; Pringle et al. 2019). Management practices associated with pheasant‐release can also impact ecosystems: the average shoot provisions 24 t of post‐release supplementary food for pheasants annually (Larkman and Newton 2015), a resource often utilised by non‐target species (Sánchez‐García et al. 2015; Willmer and Littlemore 2012). Any of these mechanisms could influence the composition of native *Borrelia* spp. host communities.

Although comparing natural *Borrelia* spp. host community composition at control and release woods was beyond the scope of the present study, we argue that direct pathogen amplification by pheasants (i.e., direct spillback) is the most parsimonious explanation for the observed *Borrelia* sp. amplification at pheasant‐release woods. *B. garinii* is both the genospecies that most frequently infects pheasants (unpublished data E.M.) and the genospecies most affected by pheasant‐release. *Borrelia garinii* amplification could potentially result from an increase in the abundance of other bird species at pheasant‐release woods. However, prior work suggests that whereas the abundance of some bird species is increased at pheasant‐release woods (e.g., woodpigeons [ *Columba palumbus* Linnaeus 1758]), there does not seem to be an effect on the abundance of typical *B. garinii* hosts (Taragel'ová et al. 2008), such as thrushes (*Turdus* spp. Linnaeus 1758) (Draycott et al. 2008). Notably, even at small shoots, the biomass of released pheasants is orders of magnitude greater than that of native avian hosts (Newson et al. 2005). Furthermore, though previous work has observed positive associations between bank vole ( *Myodes glareolus* Schreber 1780) and wood mouse ( *Apodemus sylvaticus* Linnaeus 1758) abundances and year‐round pheasant food supplementation (Davey 2008), *B. afzelii*, which typically infects small mammals (Hanincová et al. 2003), was not amplified in release woods in our study, suggesting that increased rodent abundance is unlikely to drive *Borrelia* spp. amplification. Nonetheless, confirming the mechanisms through which pheasant‐release amplifies *Borrelia* spp. prevalence would be an important next step to identify intervention strategies for the mitigation of the impact of non‐natives on zoonotic disease risk.

Currently the spatial extent of pheasant‐release effects on Lyme disease risk amplification is unclear. We show that *Borrelia* spp. prevalence in ticks was almost 2.5x higher in woods where pheasants are released compared to control woods 1–2.5 km away, a range based on previously reported pheasant dispersal distances. However, in contexts that promote greater dispersal distances‐such as low food availability (Kreuzinger‐Janik et al. 2022) or high connectivity (Baguette and Van Dyck 2007)—pheasants could potentially move further away from release sites. As such, *Borrelia* spp. prevalence might continue to decrease beyond 1–2.5 km distance from release pens. Whether the effects of pheasant‐release are localised or extend gradually across the landscape will determine if Lyme disease risk amplification primarily represents an occupational health hazard to gamebird managers exposed to infected ticks during pheasant husbandry, or a broader health hazard to the general public.

In conclusion, we show that the release of non‐native pheasants for recreational shooting is associated with an almost 2.5× higher prevalence of *Borrelia* spp., the causative agent of Lyme disease, in questing ticks. The fact that this effect is primarily driven by the amplification of the bird specialist *B. garinii*, together with the replicated, quasi‐experimental design of our study, provides the strongest evidence to date that non‐native species can impact zoonotic pathogen prevalence via spillback in ecologically relevant contexts.

## Author Contributions

Conceptualisation and study design: E.M., B.T., J.M.M., R.A.M., S.E.P. Data collection: E.M., K.H. Data analysis: E.M. Writing: E.M., B.T. with feedback from J.M.M., R.A.M., S.E.P., K.H.

## Conflicts of Interest

The authors declare no conflicts of interest.

### Peer Review

The peer review history for this article is available at https://www.webofscience.com/api/gateway/wos/peer‐review/10.1111/ele.70115.

## Supporting information


Data S1.

## Data Availability

All data and code are available on Dryad and Zenodo, respectively: https://doi.org/10.5061/dryad.d7wm37q9d and https://doi.org/10.5281/zenodo.15011644.

## References

[ele70115-bib-0001] Aebischer, N. J. 2019. “Fifty‐Year Trends in UK Hunting Bags of Birds and Mammals, and Calibrated Estimation of National bag Size, Using GWCT'S National Gamebag Census.” European Journal of Wildlife Research 65, no. 4: 64.

[ele70115-bib-0002] Ayala, A. J. , M. J. Yabsley , and S. M. Hernandez . 2020. “A Review of Pathogen Transmission at the Backyard Chicken–Wild Bird Interface.” Frontiers in Veterinary Science 7: 539925. 10.3389/fvets.2020.539925. 33195512 PMC7541960

[ele70115-bib-0003] Baguette, M. , and H. Van Dyck . 2007. “Landscape Connectivity and Animal Behavior: Functional Grain as a Key Determinant for Dispersal.” Landscape Ecology 22: 1117–1129.

[ele70115-bib-0004] Balmelli, T. , and J. C. Piffaretti . 1995. “Association Between Different Clinical Manifestations of Lyme Disease and Different Species of *Borrelia burgdorferi Sensu Lato* .” Research in Microbiology 146, no. 4: 329–340. 10.1016/0923-2508(96)81056-4. 7569327

[ele70115-bib-0005] Baranton, G. U. Y. , D. Postic , I. Saint Girons , et al. 1992. “Delineation of *Borrelia Burgdorferi* Sensu Stricto, *Borrelia garinii* sp. nov., and Group VS461 Associated With Lyme Borreliosis.” International Journal of Systematic and Evolutionary Microbiology 42, no. 3: 378–383. 10.1099/00207713-42-3-378 1380285

[ele70115-bib-0006] Bezerra‐Santos, M. A. , F. Dantas‐Torres , J. A. Mendoza‐Roldan , R. C. A. Thompson , D. Modry , and D. Otranto . 2023. “Invasive Mammalian Wildlife and the Risk of Zoonotic Parasites.” Trends in Parasitology 39, no. 9: 786–798. 37429777 10.1016/j.pt.2023.06.004

[ele70115-bib-0007] Blackburn, T. M. , and K. J. Gaston . 2021. “Contribution of Non‐Native Galliforms to Annual Variation in Biomass of British Birds.” Biological Invasions 23, no. 5: 1549–1562. 10.1007/s10530-021-02458-y.

[ele70115-bib-0008] Bouwmeester, M. M. , M. A. Goedknegt , R. Poulin , and D. W. Thieltges . 2021. “Collateral Diseases: Aquaculture Impacts on Wildlife Infections.” Journal of Applied Ecology 58: 453–464.

[ele70115-bib-0009] Burkett‐Cadena, N. D. , E. M. Blosser , A. A. Loggins , et al. 2021. “Invasive Burmese Pythons Alter Host Use and Virus Infection in the Vector of a Zoonotic Virus.” Community Biology 4, no. 1: 804. 10.1038/s42003-021-02347-z PMC8239020 34183751

[ele70115-bib-0010] Bürkner, P. 2021. “Bayesian Item Response Modeling in R With Brms and Stan.” Journal of Statistical Software 100, no. 5: 1–54.

[ele70115-bib-0011] Canica, M. M. , F. Nato , L. D. Merle , J. C. Mazie , G. Baranton , and D. Postic . 1993. “Monoclonal Antibodies for Identification of *Borrelia afzelii* sp. nov. Associated With Late Cutaneous Manifestations of Lyme Borreliosis.” Scandinavian Journal of Infectious Diseases 25, no. 4: 441–448. 10.3109/00365549309008525. 8248743

[ele70115-bib-0012] Carlson, C. J. , G. F. Albery , C. Merow , et al. 2022. “Climate Change Increases Cross‐Species Viral Transmission Risk.” Nature 607, no. 7919: 555–562. 10.1038/s41586-022-04788-w. 35483403

[ele70115-bib-0013] Carpio, A. J. , J. Guerrero‐Casado , J. A. Barasona , et al. 2017. “Hunting as a Source of Alien Species: A European Review.” Biological Invasions 19, no. 4: 1197–1211.

[ele70115-bib-0014] Craine, N. G. , P. A. Nuttall , A. C. Marriott , and S. E. Randolph . 1997. “Role of Grey Squirrels and Pheasants in the Transmission of *Borrelia Burgdorferi Sensu Lato*, the Lyme Disease Spirochaete, in the U.K.” Folia Parasitologica 44, no. 2: 155–160. 9269722

[ele70115-bib-0015] Craine, N. G. , S. E. Randolph , and P. A. Nuttall . 1995. “Seasonal Variation in the Role of Grey Squirrels as Hosts of *Ixodes Ricinus*, the Tick Vector of the Lyme Disease Spirochaete, in a British Woodland.” Folia Parasitologica 42, no. 1: 73–80. 9599431

[ele70115-bib-0016] Davey, C. M. 2008. “The Impact of Game Management for Pheasant (*Phasianus colchicus*) Shooting on Vertebrate Biodiversity in British Woodlands.” Doctoral Dissertation, University of Bristol.

[ele70115-bib-0017] Downs, C. J. , L. A. Schoenle , B. A. Han , J. F. Harrison , and L. B. Martin . 2019. “Scaling of Host Competence.” Trends in Parasitology 35, no. 3: 182–192. 10.1016/j.pt.2018.12.002. 30709569

[ele70115-bib-0018] Draycott, R. A. H. , A. N. Hoodless , and R. B. Sage . 2008. “Effects of Pheasant Management on Vegetation and Birds in Lowland Woodlands.” Journal of Applied Ecology 45: 334–341.

[ele70115-bib-0019] Draycott, R. A. H. , M. I. A. Woodburn , D. E. Ling , and R. B. Sage . 2006. “The Effect of an Indirect Anthelmintic Treatment on Parasites and Breeding Success of Free‐Living Pheasants (*Phasianus colchicus*).” Journal of Helminthology 80, no. 4: 409–415. 17125551 10.1017/joh2006367

[ele70115-bib-0080] Faust, C. L. , H. I. McCallum , L. S. P. Bloomfield , et al. 2018. “Pathogen Spillover During Land Conversion.” Ecology Letters 21, no. 4: 471–483. 10.1111/ele.12904. 29466832

[ele70115-bib-0020] Fracasso, G. , M. Grillini , L. Grassi , F. Gradoni , G. D. Rold , and M. Bertola . 2023. “Effective Methods of Estimation of Pathogen Prevalence in Pooled Ticks.” Pathogens 12, no. 4: 557. 10.3390/pathogens12040557. 37111443 PMC10146257

[ele70115-bib-0081] Gottdenker, N. L. , D. G. Streicker , C. L. Faust , and C. R. Carroll . 2014. “Anthropogenic Land Use Change and Infectious Diseases: A Review of the Evidence.” EcoHealth 11, no. 4: 619–632. 10.1007/s10393-014-0941-z. 24854248

[ele70115-bib-0021] Halliday, F. W. , J. R. Rohr , and A.‐L. Laine . 2020. “Biodiversity Loss Underlies the Dilution Effect of Biodiversity.” Ecology Letters 23, no. 11: 1611–1622. 10.1111/ele.13590. 32808427 PMC7693066

[ele70115-bib-0022] Hanincová, K. , S. M. Schäfer , S. Etti , et al. 2003. “Association of *Borrelia afzelii* With Rodents in Europe.” Parasitology 126, no. 1: 11–20. 10.1017/s0031182002002548. 12613759

[ele70115-bib-0023] Hansford, K. M. , M. Fonville , E. L. Gillingham , et al. 2017. “Ticks and *Borrelia* in Urban and Peri‐Urban Green Space Habitats in a City in Southern England.” Ticks and Tick‐Borne Diseases 8, no. 3: 353–361. 28089123 10.1016/j.ttbdis.2016.12.009

[ele70115-bib-0024] Hansford, K. M. , M. Fonville , A. Jahfari , S. Jahfari , H. Sprong , and J. M. Medlock . 2015. “ *Borrelia miyamotoi* in Host‐Seeking *Ixodes ricinus* Ticks in England.” Epidemiology and Infection 143, no. 5: 1079–1087. 10.1017/S0950268814001691. 25017971 PMC9507118

[ele70115-bib-0025] Hansford, K. M. , L. McGinley , B. W. Wheeler , et al. 2023. “ *Ixodes Ricinus* Density, *Borrelia* Prevalence and the Density of Infected Nymphs Along an Rrban‐Rural Gradient in Southern England.” Zoonoses and Public Health 70, no. 4: 304–314. 36660965 10.1111/zph.13024

[ele70115-bib-0026] Hansford, K. M. , N. T. Wheeler , B. Tschirren , B. W. Wheeler , B. Tshirren , and J. M. Medlock . 2022. “Urban Woodland Habitat Is Important for Tick Presence and Density in a City in England.” Ticks and Tick‐Borne Diseases 13, no. 1: 101857. 10.1016/j.ttbdis.2021.101857. 34763308

[ele70115-bib-0027] Hillyard, P. D. 1996. Ticks of North‐West Europe: Keys and Notes for Identification of the Species. Field Studies Council, Linnean Society of London and the Estuarine and Coastal Sciences Association.

[ele70115-bib-0076] Hoodless, A. N. , K. Kurtenbach , P. A. Nuttall , and S. E. Randolph . 2002. “The Impact of Ticks on Pheasant Territoriality.” Oikos 96, no. 2: 245–250. 10.1034/j.1600-0706.2002.960206.x.

[ele70115-bib-0028] Hoyer, I. J. , E. M. Blosser , C. Acevedo , A. C. Thompson , L. E. Reeves , and N. D. Burkett‐Cadena . 2017. “Mammal Decline, Linked to Invasive Burmese Python, Shifts Host Use of Vector Mosquito Towards Reservoir Hosts of a Zoonotic Disease.” Biology Letters 13, no. 10: 20170353. 10.1098/rsbl.2017.0353. 28978755 PMC5665769

[ele70115-bib-0029] Jeschke, J. M. , and D. L. Strayer . 2006. “Determinants of Vertebrate Invasion Success in Europe and North America.” Global Change Biology 12, no. 9: 1608–1619. 10.1111/j.1365-2486.2006.01213.x.

[ele70115-bib-0030] Johnson, R. C. , G. P. Schmid , F. W. Hyde , A. G. Steigerwalt , and D. J. Brenner . 1984. “ *Borrelia burgdorferi* sp. nov.: Etiologic Agent of Lyme Disease.” International Journal of Systematic Bacteriology 34, no. 4: 496–497. 10.1099/00207713-34-4-496.

[ele70115-bib-0031] Kahl, O. , and J. S. Gray . 2023. “The Biology of *Ixodes Ricinus* With Emphasis on Its Ecology.” Ticks and Tick‐Borne Diseases 14, no. 2: 102114. 36603231 10.1016/j.ttbdis.2022.102114

[ele70115-bib-0032] Kelly, D. W. , R. A. Paterson , C. R. Townsend , R. Poulin , and D. M. Tompkins . 2009. “Parasite Spillback: A Neglected Concept in Invasion Ecology?” Ecology 90, no. 8: 2047–2056. 19739367 10.1890/08-1085.1

[ele70115-bib-0033] Kennedy, T. A. , S. Naeem , K. H. Howe , et al. 2002. “Biodiversity as a Barrier to Ecological Invasion.” Nature 417, no. 6889: 636–638. 10.1038/nature00776. 12050662

[ele70115-bib-0034] Kreuzinger‐Janik, B. , B. Gansfort , W. Traunspurger , and C. Ptatscheck . 2022. “It's all About Food: Environmental Factors Cause Species‐Specific Dispersal.” Ecosphere 13, no. 10: e4251.

[ele70115-bib-0035] Kurtenbach, K. , D. Carey , A. N. Hoodless , P. A. Nuttall , and S. E. Randolph . 1998. “Competence of Pheasants as Reservoirs for Lyme Disease Spirochetes.” Journal of Medical Entomology 35, no. 1: 77–81. 9542349 10.1093/jmedent/35.1.77

[ele70115-bib-0036] Kurtenbach, K. , M. Peacey , S. G. Rijpkema , A. N. Hoodless , P. A. Nuttall , and S. E. Randolph . 1998. “Differential Transmission of the Genospecies of *Borrelia burgdorferi Sensu Lato* by Game Birds and Small Rodents in England.” Applied and Environmental Microbiology 64, no. 4: 1169–1174. 10.1128/AEM.64.4.1169-1174.1998. 9546150 PMC106125

[ele70115-bib-0037] Larkman, A. , and I. Newton . 2015. “Small Farmland Bird Declines, Gamebird Releases, and Changes in Seed Sources.” In Wildlife Conservation on Farmland. Conflict in the Countryside, edited by D. W. Macdonald and R. E. Feber , 181–202. Oxford University Press.

[ele70115-bib-0038] Lindgren, E. , and T. G. T. Jaenson . 2006. Lyme Borreliosis in Europe: Influences of Climate and Climate Change, Epidemiology, Ecology and Adaptation Measures. World Health Organization, Regional Office for Europe. https://iris.who.int/handle/10665/107800.

[ele70115-bib-0039] MacDougall, A. S. , J. R. Bennett , J. Firn , et al. 2014. “Anthropogenic‐Based Regional‐Scale Factors Most Consistently Explain Plot‐Level Exotic Diversity in Grasslands.” Global Ecology and Biogeography 23, no. 7: 802–810.

[ele70115-bib-0040] Macleod, J. 1936. “ *Ixodes Ricinus* in Relation to Its Physical Environment: IV. An Analysis of the Ecological Complexes Controlling Distribution and Activities.” Parasitology 28, no. 3: 295–319. 10.1017/S0031182000022502.

[ele70115-bib-0041] Madden, J. R. , R. Buckley , and S. Ratcliffe . 2023. “Large‐Scale Correlations Between Gamebird Release and Management and Animal Biodiversity Metrics in Lowland Great Britain.” Ecology and Evolution 13, no. 5: e10059. 37168985 10.1002/ece3.10059 PMC10166649

[ele70115-bib-0042] Madden, J. R. , and R. B. Sage . 2020. Ecological Consequences of Gamebird Releasing and Management on Lowland Shoots in England: A Review by Rapid Evidence Assessment for Natural England and the British Association of Shooting and Conservation. Natural England.

[ele70115-bib-0043] Marsot, M. , J.‐L. Chapuis , P. Gasqui , et al. 2013. “Introduced Siberian Chipmunks (* Tamias sibiricus Barberi*) Contribute More to Lyme Borreliosis Risk Than Native Reservoir Rodents.” PLoS One 8, no. 1: e55377. 23383170 10.1371/journal.pone.0055377 PMC3561227

[ele70115-bib-0044] Mbora, D. N. M. , and M. A. McPeek . 2009. “Host Density and Human Activities Mediate Increased Parasite Prevalence and Richness in Primates Threatened by Habitat Loss and Fragmentation.” Journal of Animal Ecology 78, no. 1: 210–218. 19120603 10.1111/j.1365-2656.2008.01481.x

[ele70115-bib-0045] McElreath, R. 2020. Statistical Rethinking: A Bayesian Course With Examples in R and Stan. 2nd ed. Chapman and Hall/CRC.

[ele70115-bib-0046] Medlock, J. M. , A. G. C. Vaux , S. Gandy , et al. 2022. “Spatial and Temporal Heterogeneity of the Density of *Borrelia burgdorferi* ‐Infected *Ixodes ricinus* Ticks Across a Landscape: A 5‐Year Study in Southern England.” Medical and Veterinary Entomology 36, no. 3: 356–370. 35521893 10.1111/mve.12574 PMC9545817

[ele70115-bib-0047] Nanetti, A. , L. Bortolotti , and G. Cilia . 2021. “Pathogens Spillover From Honey Bees to Other Arthropods.” Pathogens 10, no. 8: 1044. 34451508 10.3390/pathogens10081044 PMC8400633

[ele70115-bib-0048] Narayan, E. 2019. “Physiological Stress Levels in Wild Koala Sub‐Populations Facing Anthropogenic Induced Environmental Trauma and Disease.” Scientific Reports 9, no. 1: 6031. 30988329 10.1038/s41598-019-42448-8 PMC6465306

[ele70115-bib-0049] Nelson, C. , S. Banks , C. L. Jeffries , T. Walker , and J. G. Logan . 2015. “Tick Abundances in South London Parks and the Potential Risk for Lyme Borreliosis to the General Public.” Medical and Veterinary Entomology 29, no. 4: 448–452. 10.1111/mve.12137. 26400641

[ele70115-bib-0050] Neumann, J. L. , G. J. Holloway , R. B. Sage , and A. N. Hoodless . 2015. “Releasing of Pheasants for Shooting in the UK Alters Woodland Invertebrate Communities.” Biological Conservation 191: 50–59.

[ele70115-bib-0051] Newson, S. E. , R. J. W. Woodburn , D. G. Noble , S. R. Baillie , and R. D. Gregory . 2005. “Evaluating the Breeding Bird Survey for Producing National Population Size and Density Estimates.” Bird Study 52, no. 1: 42–54. 10.1080/00063650509461373.

[ele70115-bib-0052] Ostfeld, R. S. 2009. “Biodiversity Loss and the Rise of Zoonotic Pathogens.” Clinical Microbiology and Infection 15, no. 1: 40–43. 19220353 10.1111/j.1469-0691.2008.02691.x

[ele70115-bib-0053] Parola, P. , G. Diatta , C. Socolovschi , et al. 2011. “Tick‐Borne Relapsing Fever Borreliosis, Rural Senegal.” Emerging Infectious Diseases 17, no. 5: 883–885. 21529402 10.3201/eid1705.100573 PMC3321757

[ele70115-bib-0079] Peter, N. , D. D. Dörge , S. Cunze , et al. 2023. “Raccoons Contraband – The Metazoan Parasite Fauna of Free‐Ranging Raccoons in Central Europe.” International Journal for Parasitology: Parasites and Wildlife 20: 79–88. 10.1016/j.ijppaw.2023.01.003. 36688078 PMC9852791

[ele70115-bib-0054] Power, A. G. , and C. E. Mitchell . 2004. “Pathogen Spillover in Disease Epidemics.” American Naturalist 164, no. 5: 79–89. 10.1086/424610 15540144

[ele70115-bib-0055] Pringle, H. , M. Wilson , J. Calladine , and G. Siriwardena . 2019. “Associations Between Gamebird Releases and Generalist Predators.” Journal of Applied Ecology 56, no. 8: 2102–2113. 10.1111/1365-2664.13451.

[ele70115-bib-0056] QGIS Association . n.d. “QGIS.” https://qgis.org/en/site/.

[ele70115-bib-0057] R Core Team . 2023. “R: A Language and Environment for Statistical Computing.” https://www.r‐project.org/.

[ele70115-bib-0058] Richter, D. , A. Debski , Z. Hubalek , and F. R. Matuschka . 2012. “Absence of Lyme Disease Spirochetes in Larval *Ixodes ricinus* Ticks.” Vector‐Borne and Zoonotic Diseases 12, no. 1: 21–27. 21923267 10.1089/vbz.2011.0668

[ele70115-bib-0059] Rollend, L. , D. Fish , and J. E. Childs . 2013. “Transovarial Transmission of *Borrelia* Spirochetes by *Ixodes Scapularis*: A Summary of the Literature and Recent Observations.” Ticks and Tick‐Borne Diseases 4, no. 1‐2: 46–51. 10.1016/j.ttbdis.2012.06.008. 23238242

[ele70115-bib-0060] Roy, H. E. , E. Tricarico , R. Hassall , et al. 2023. “The Role of Invasive Alien Species in the Emergence and Spread of Zoonoses.” Biological Invasions 25, no. 4: 1249–1264. 36570096 10.1007/s10530-022-02978-1 PMC9763809

[ele70115-bib-0061] Sage, R. B. , M. I. A. Woodburn , R. A. H. Draycott , A. N. Hoodless , and S. Clarke . 2009. “The Flora and Structure of Farmland Hedges and Hedgebanks Near to Pheasant Release Pens Compared With Other Hedges.” Biological Conservation 142, no. 7: 1362–1369. 10.1016/j.biocon.2009.01.034.

[ele70115-bib-0062] Sage, R. R. , C. Ludolf , and P. A. Robertson . 2005. “The Ground Flora of Ancient Semi‐Natural Woodlands in Pheasant Release Pens in England.” Biological Conservation 122, no. 2: 243–252.

[ele70115-bib-0063] Sánchez‐García, C. , F. D. Buner , and N. J. Aebischer . 2015. “Supplementary Winter Food for Gamebirds Through Feeders: Which Species Actually Benefit?” Journal of Wildlife Management 79, no. 5: 832–845.

[ele70115-bib-0064] Sardain, A. , E. Sardain , and B. Leung . 2019. “Global Forecasts of Shipping Traffic and Biological Invasions to 2050.” Nature Sustainability 2, no. 4: 274–282. 10.1038/s41893-019-0245-y.

[ele70115-bib-0065] Stachowicz, J. J. , H. Fried , R. W. Osman , and R. B. Whitlatch . 2002. “Biodiversity, Invasion Resistance, and Marine Ecosystem Function: Reconciling Pattern and Process.” Ecology 83, no. 9: 2575–2590.

[ele70115-bib-0077] Stan Development Team . 2023. “Stan: A Probabilistic Programming Language.” Version 2.x. https://mc‐stan.org.

[ele70115-bib-0078] Stefanski, W. , and E. Zarnowski . 1951. “*Ascaris procyonis* n. sp. From the Intestine of *Procyon lotor* L.”

[ele70115-bib-0066] Swei, A. C. , J. Briggs , R. S. Lane , A. Swei , C. J. Briggs , and R. S. Ostfeld . 2012. “Impacts of an Introduced Forest Pathogen on the Risk of Lyme Disease in California.” Vector‐Borne and Zoonotic Diseases 12, no. 8: 623–632. 10.1089/vbz.2011.0783. 22607076 PMC3413889

[ele70115-bib-0067] Taragel'ová, V. , J. Koci , K. Hanincová , et al. 2008. “Blackbirds and Song Thrushes Constitute a Key Reservoir of *Borrelia garinii*, the Causative Agent of Borreliosis in Central Europe.” Applied and Environmental Microbiology 74, no. 4: 1289–1293. 18156328 10.1128/AEM.01060-07 PMC2258561

[ele70115-bib-0068] Turner, C. V. 2008. “The Fate and Management of Pheasants (*Phasianus colchicus*) Released in the UK.” Doctoral dissertation, Imperial College London.

[ele70115-bib-0069] Umhang, G. , C. Richomme , J.‐M. Boucher , G. Guedon , and F. Boué . 2013. “Nutrias and Muskrats as Bioindicators for the Presence of *Echinococcus multilocularis* in New Endemic Areas.” Veterinary Parasitology 197, no. 1‐2: 283–287. 23725822 10.1016/j.vetpar.2013.05.003

[ele70115-bib-0070] van Dam, A. P. , H. Kuiper , K. Vos , et al. 1993. “Different Genospecies of *Borrelia burgdorferi* Are Associated With Distinct Clinical Manifestations of Lyme Borreliosis.” Clinical Infectious Diseases 17, no. 4: 708–717. 7903558 10.1093/clinids/17.4.708

[ele70115-bib-0072] Wang, G. , A. P. Van Dam , A. L. Fleche , et al. 1997. “Genetic and Phenotypic Analysis of *Borrelia valaisiana* sp. nov.(Borrelia Genomic Groups VS116 and M19).” International Journal of Systematic and Evolutionary Microbiology 47, no. 4: 926–932. 10.1099/00207713-47-4-926 9336888

[ele70115-bib-0073] Willmer, S. , and J. Littlemore . 2012. “Gamebird Feeding Hoppers Provide Winter Food for Non‐target Wildlife Species Including Declining Songbirds on Small Farms in the East Midlands, England.” Accessed 01 10 2024. http://www.rectory‐farm.org.uk/assets/gamebird‐feeding‐hoppers.pdf.

[ele70115-bib-0074] Young, H. S. , I. M. Parker , G. S. Gilbert , et al. 2017. “Introduced Species, Disease Ecology, and Biodiversity‐Disease Relationships.” Trends in Ecology and Ecolution 32, no. 1: 41–54. 10.1016/j.tree.2016.09.008 28029377

[ele70115-bib-0075] Zhang, L. , J. Rohr , R. Cui , et al. 2022. “Biological Invasions Facilitate Zoonotic Disease Emergences.” Nature Communications 13, no. 1: 1762. 10.1038/s41467-022-29378-2. PMC8975888 35365665

