# Supplementary material for: The Release of Non‐Native Gamebirds Is Associated With Amplified Zoonotic Disease Risk
Source: Ecol Lett. 2025 Apr 21;28(4):e70115. doi: 10.1111/ele.70115 (PMC12010324; doi:10.1111/ele.70115)
Supplement: Supplementary file 1 — Data S1. [file ELE-28-0-s001.docx]

**Supplementary information for “The release of non-native gamebirds is associated with amplified zoonotic disease risk”**

Emile Michels^1*^, Kayleigh Hansford^2^, Sarah E. Perkins^3^, Robbie A. McDonald^1^,^4^, Jolyon M. Medlock^2^, Barbara Tschirren^1^

1. University of Exeter, Centre for Ecology and Conservation, Penryn, Cornwall, TR10 9FE, UK
2. Medical Entomology and Zoonoses Ecology Group, UK Health Security Agency, Porton Down, Salisbury, SP4 0JG, UK
3. School of Biosciences, Cardiff University, Sir Martin Evans Building, Cardiff CF10 3AX, UK
4. University of Exeter, Environment and Sustainability Institute, University of Exeter, Penryn, Cornwall, TR10 9FE, UK

*Corresponding author; email: emile.michels@hotmail.co.uk University of Exeter, Centre for Ecology and Conservation, Penryn, Cornwall, TR10 9FE, UK

**Supplementary Methods 1 - Estimating tick *Borrelia* prevalence from pooled samples**

As ~93% of ticks were pooled (i.e. 2 ticks analysed together) for DNA extraction and *Borrelia sp.* testing, it was not possible to establish the ‘true’ *Borrelia* prevalence among the ticks sampled. However, by using a misclassification or measurement error model [(Abel et al., 1999, Scelza et al., 2020)](https://paperpile.com/c/a5NgFR/D5FT), we acquired prevalence estimates which can be compared with prevalence estimates in existing literature where no pooling has taken place. Furthermore, as 1/203 of the extraction controls used during qPCR tested positive for *Borrelia sp.*, this suggests that some sample cross-contamination could have taken place during DNA extraction. As such, the model likelihood also incorporated a 0.05% false positive rate. As such, the likelihoods used to calculate *Borrelia sp.* prevalence in ticks are as follows.

For a single positive tick: *p*i + (1 - *p*i) * *f*

For a single negative tick: 1 - *p*i * (1 - *f*)

For a pool of two ticks which tests *Borrelia sp.* negative: (1 - *p*i)^2^ * 1 - *f*

For a pool of two ticks which tests *Borrelia sp.* positive: *p*i + (1 - *p*i)*p*i + ((1 - *p*i)^2^)*f*

Where *Borrelia sp.* prevalence is ‘*p*’ and the probability of a false positive ‘*f’* is 0.05%.

For comparison, the likelihoods used in a standard Binomial model with no misclassification component would be written as follows:

For a single positive tick: *p*i

For a single negative tick: 1 - *p*i

An advantage of using such a misclassification model is that the estimated probability of both ticks in a pool being simultaneously infected is not fixed, but changes in response to the estimated prevalence across all pools from a given wood and shoot, i.e. if many pools from one specific wood are positive, then the probability that both of the ticks in a positive pool from that wood are simultaneously positive is higher. Hence, wood and shoot level random effects, as well as other fixed variables, can correctly be incorporated into prevalence estimates.

**Supplementary results 1 - Ecological null model selection**


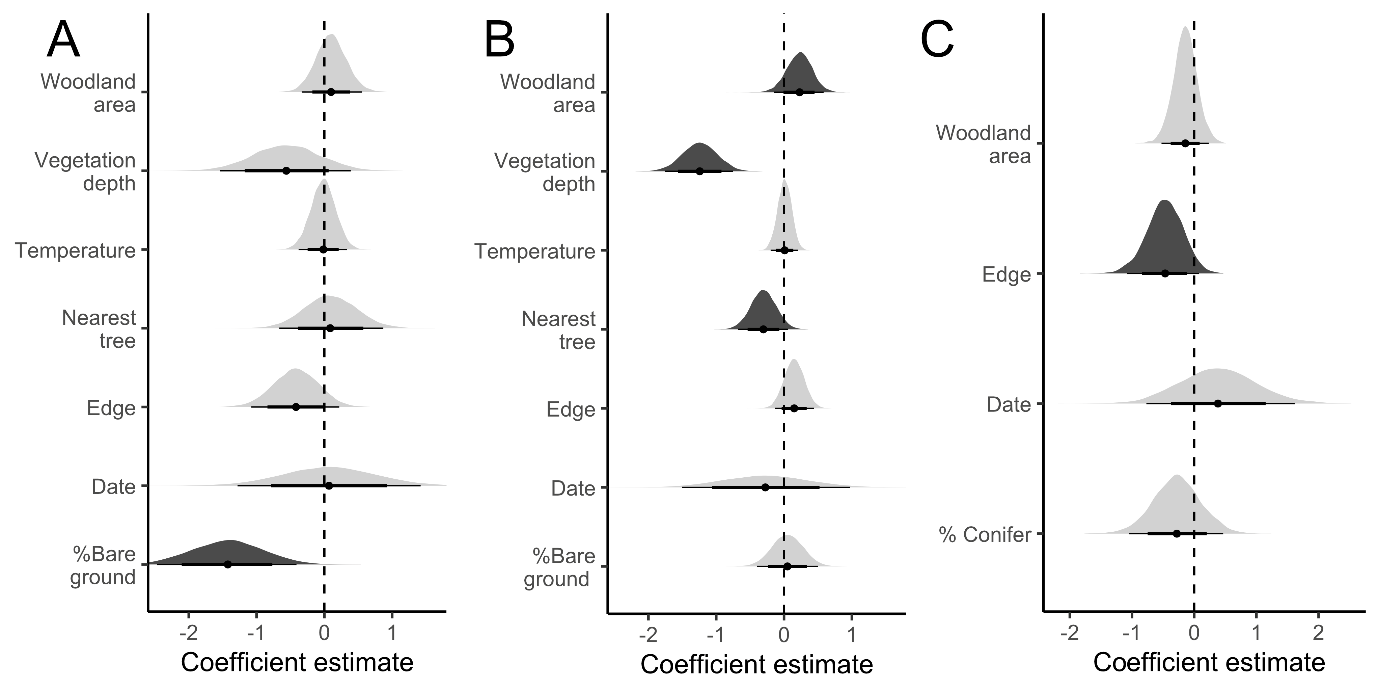


**Supplementary FIGURE S1**

The posterior distributions for all variables included in the statistical exploration used to determine which ecological parameters to include in the ecological null models for A) adult tick abundance, B) nymphal tick abundance and C) tick *Borrelia* sp. prevalence. Mean coefficient estimates are represented by black points, horizontal black lines under each distribution represent 79% and 95% highest probability density intervals. All variables retained in the ecological null model are filled in dark grey. For ‘woodland area’ positive values indicate that tick abundance increases with the continuous size of woodlands. For ‘vegetation depth’, negative values indicate that tick abundance decreases as vegetation depth increases. For ‘nearest tree’, negative values indicate that tick abundance is higher if the tree nearest to where a drag took place was a conifer. For ‘bare ground’ negative values indicate that tick abundance decreases as the percentage of ground covered with understory vegetation decreases. For ‘edge’, negative values indicate that tick *Borrelia sp.* prevalence is higher at the edge of woods. Variables measured for each drag (e.g. vegetation depth, bare ground percent cover) were not considered in *Borrelia* sp. prevalence model selection because the drags which individuals ticks came from was not recorded.

**Supplementary results 2 – ecological null model sensitivity analyses**

To assess if systematic differences between control and release woods influence tick abundance and *Borrelia* sp. prevalence independently of pheasant release, we tested for differences between control and release woods regarding the variables included in our ecological null models. Furthermore, to evaluate whether the estimated effects of pheasant-release on tick abundance and *Borrelia* sp. prevalence depend on the inclusion of specific ecological variables in the models, we re-ran all models with pheasant-release as a predictor, iteratively removing all combinations of ecological variables.

We observed no differences between control and release woods regarding %bare ground cover (mean effect: -0.06, 95% HPDI: -0.39 to 0.26 (logit scale)), vegetation depth (mean effect: 0.03, 95% HPDI: -0.29 to 0.37 (logit scale)), proportion of edge habitat (mean effect: -0.52, 95% HPDI: -1.26 to 0.20 (logit scale)) or woodland area (mean effect: 0.08, 95% HPDI: -0.05 to 0.2 (standardised scale)). Conifer trees were more common at pheasant release woods than control woods (mean effect: -1.66, 95% HPDI: -3.08 to -0.45 (logit scale)), but sensitivity analyses showed that the inclusion of tree type (i.e. conifer vs broadleaf) nearest to each drag in ecological null models, or the inclusion of any other ecological variables, does not alter the estimated effect size of pheasant-release on tick abundance or *Borrelia* sp. prevalence in ticks (Supplementary Figure S2).


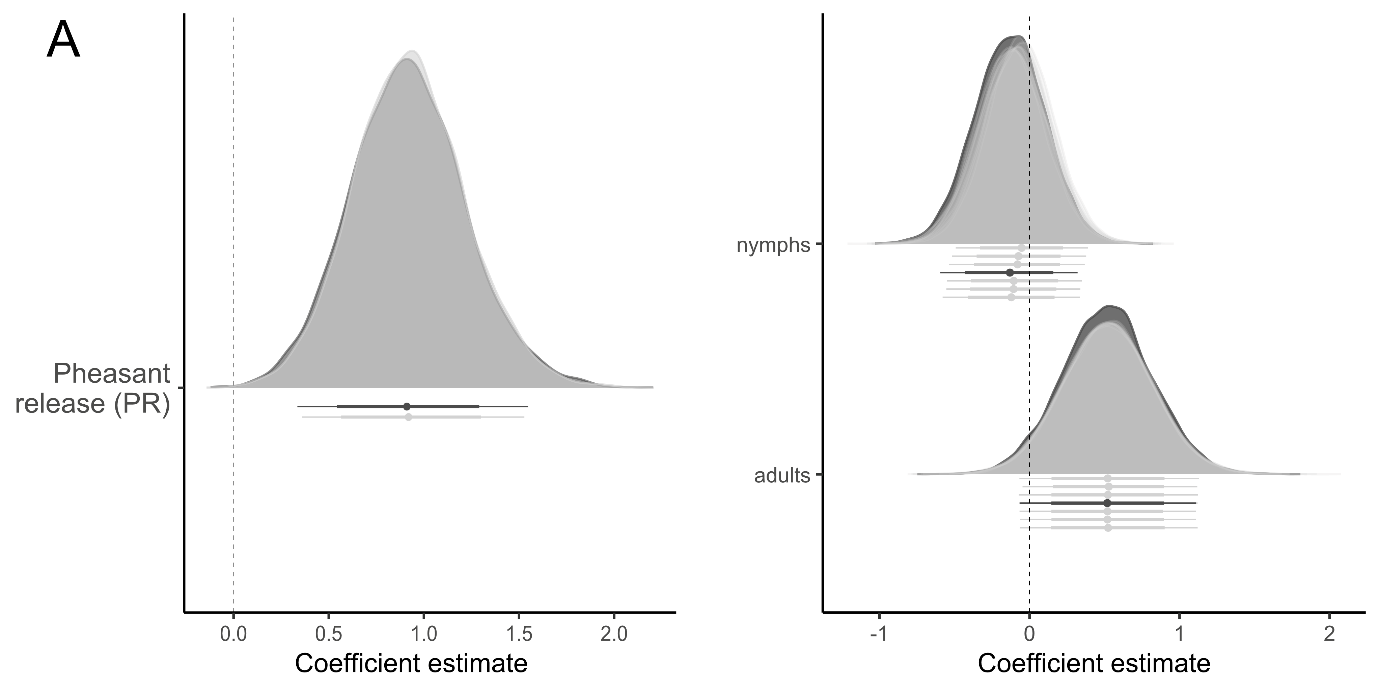


**Supplementary FIGURE S2**

The posterior distributions for the effects of pheasant-release on A) *Borrelia* sp. prevalence in ticks and B) nymphal and adult tick abundances. Posterior distributions reported in the main text are displayed in dark grey, posterior distributions from subsequent models where ecological variables are sequentially removed from the model are superimposed in light grey. Beneath each distribution mean coefficient estimates are represented by points, horizontal lines under each distribution represent 79% and 95% highest probability density intervals.

**Supplementary results 3 – release magnitude analysis**

To test if the observed effects of pheasant-release were dependant on the number of pheasants released at a shoot, we re-ran the analyses with release magnitude and a magnitude x pheasant-release interaction included as additional predictor variables in both the tick abundance, and tick *Borrelia* sp. prevalence models. Our study was not designed to test for effects of pheasant release along a continuum of release magnitudes, as such we sampled only 1 shoot which released an exceptionally high number of pheasants (i.e. 4 standard deviations more pheasants than the mean). To assess this outlier shoot’s influence on the results, we repeated the analyses excluding this extreme value.

Adding release magnitude and a release magnitude x pheasant-release interaction to models did not change our estimates for the effect of pheasant-release on *Borrelia* sp. prevalence (mean effect: 0.34, 95% HPDI: 0.96 to 1.58 (logit scale)) or the effect of pheasant-release on nymph abundance (mean effect: -0.15, 95% HPDI: -0.55 to 0.25 (log scale)). However, the estimated effect of pheasant-release on adult tick abundance did change, so that the 95% HPDI of this variable’s posterior distribution no longer spanned 0 (mean effect: 0.59, 95% HPDI: 0.05 to 1.13 (log scale)) (i.e. pheasant-release effect became greater).

We found little evidence that a magnitude x pheasant-release interaction influences tick *Borrelia* sp. prevalence (mean effect: 0.33, 95% HPDI: -0.25 to 0.91 (logit scale)). We found some evidence that adult (mean effect: -0.75, 95% HPDI: -1.27 to -0.26 (log scale)) and nymph abundances (mean effect: -0.89, 95% HPDI: -1.34 to -0.46 (log scale)) are influenced by an interaction between pheasant-release and release magnitude. These results suggest that with increased release magnitude, adult abundance in control woods increased (mean slope estimate: 0.72, 95% HPDI: 0.25 to 1.167 (log scale)), whilst nymph abundance in release woods decreased (mean slop estimate: -0.55, 95% HPDI: -0.02 to 1.03 (log scale)). Adult abundance in release woods (mean slope estimate: -0.03, 95% HPDI: -0.58 to 0.5 (log scale)) and nymph abundance in control woods (mean slope estimate; 0.35, 95% HPDI: -0.08 to 0.79 (log scale)) were unaffected by release magnitude. However, when data from an outlier shoot were removed, the 95% HPDI’s of all posterior slope estimates spanned 0 (Supplementary Figure S3), suggesting that these release magnitude effects were not robust.


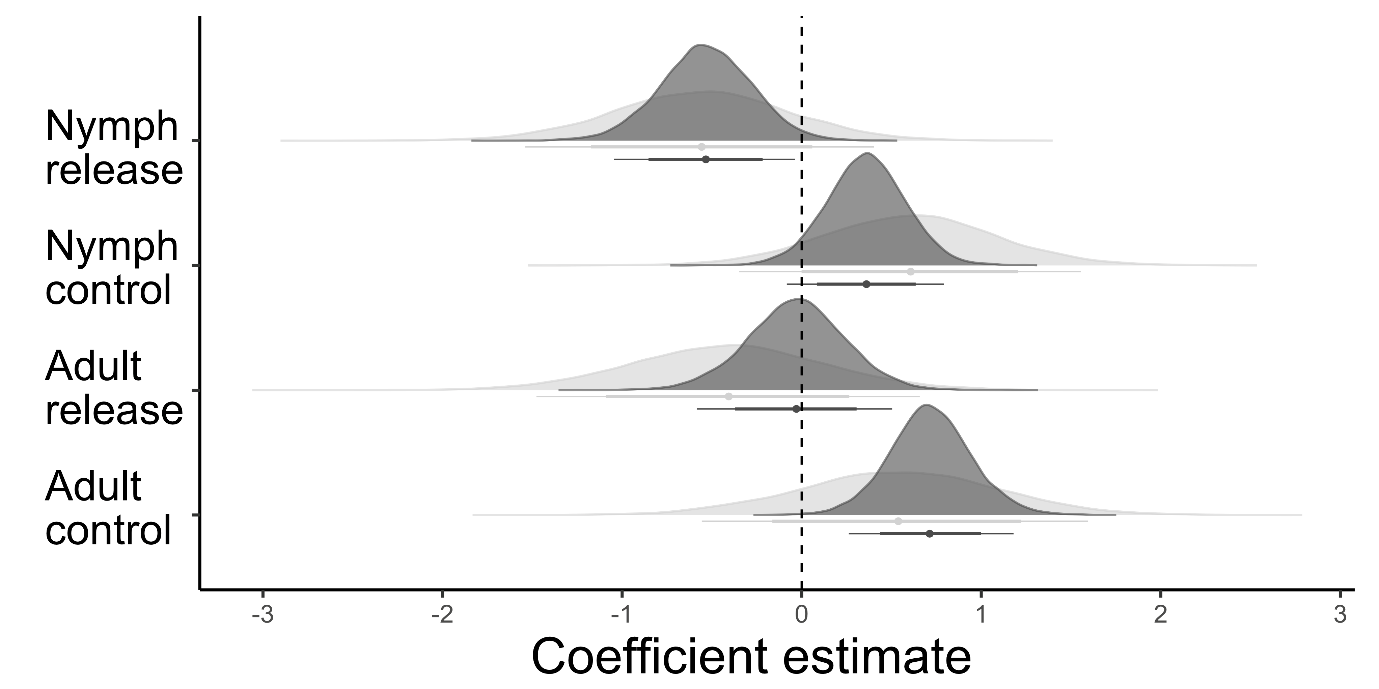


**Supplementary FIGURE S3**

Posterior distributions of the estimated slope coefficients for the relationships between tick abundance and pheasant release magnitude for, from top to bottom; nymphal tick abundance in woods where pheasants are released, nymphal tick abundance in control woods where pheasants are not released, adult tick abundance in woods where pheasants are released, adult tick abundance in control woods where no pheasants are released. Dark posterior distributions are drawn from models including all data, light-grey distributions are from models where data from one extreme shoot has been removed, a high leverage outlier where 105,000 pheasants are released.

**Supplementary results 4 – Tick abundance and *Borrelia* sp. prevalence model posterior distributions**

**
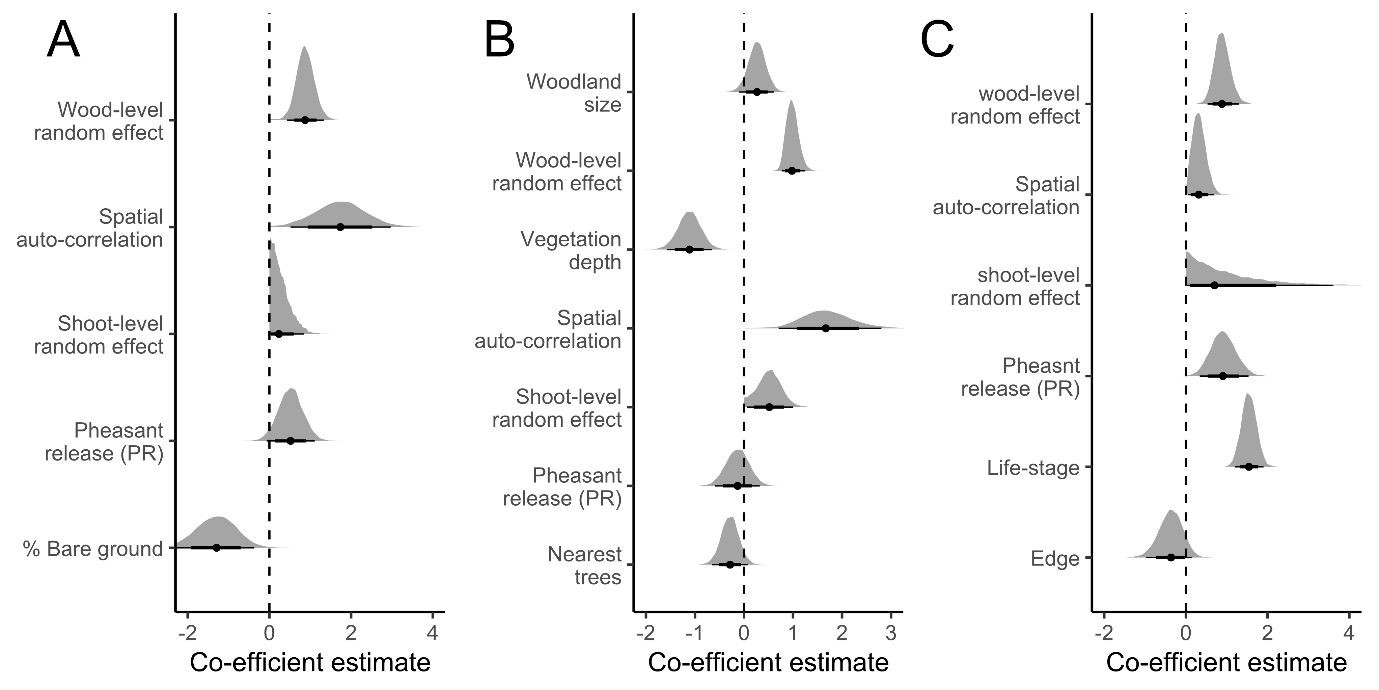
**

**Supplementary FIGURE S4**

The posterior distributions for all variables included in models predicting A) adult tick abundance, B) nymphal tick abundance and C) tick *Borrelia* sp. prevalence. Mean coefficient estimates are represented by black points, horizontal black lines under each distribution represent 79% and 95% highest probability density intervals.

**Supplementary results 5 – genospecies model posterior distribution summaries**

**Supplementary TABLE S1**

Posterior distributions for the effects of life stage, pheasant-release, and the pheasant-release x life stage interaction on the prevalence of the three most common *Borrelia* genospecies among our sample of *I. ricinus* ticks. Distributions are summarised using mean estimates and 95% highest probability density intervals (HPDI). Distributions for which 95% HPDI’s do not span 0 are highlighted in bold. All values are on the logit scale.

|  | Pheasant release | Life stage | Pheasant release x Life stage interaction |
| --- | --- | --- | --- |
| *B. afzelii* | -0.10 (-0.68 - 0.49) | 0.39 (-0.24 - 1.05) | -0.67 (-1.5 - 0.12) |
| *B. garinii* | **1.04 (0.55 - 1.52)** | **0.85 (0.45 - 1.29)** | 0.14 (-0.54 - 0.84) |
| *B. valaisiana* | **0.50 (0.00 - 1.00)** | **0.91 (0.45 - 1.37)** | **0.77 (0.06 - 1.48)** |

**Supplementary TABLE S2**

Pairwise differences in how the prevalence of the three most common *Borrelia* genospecies are affected by an interaction between pheasant-release and the life stage of *I. ricinus* ticks. Differences are calculated from pairwise contrast, i.e. subtracting the posterior distribution for a variable's effect on one genospecies, from the distribution of that same variable's effect on another genospecies. Contrasts are themselves distributions and are thus summarised using means and 95% highest probability density intervals. The pairwise contrasts for which 95% HPDI’s do not span 0 are highlighted in bold. All values are on the logit scale. See Supplementary table S1 for overall pheasant-release x life stage interactions.

|  |  | *B. garinii* | *B. afzelii* |
| --- | --- | --- | --- |
| Pheasant release | *B. garinii* |  | **1.15 (0.46 - 1.85)** |
|  | *B. valaisiana* | 0.55 (-0.03 - 1.15) | 0.61 (-0.12 - 1.32) |
| Life stage | *B. garinii* |  | 0.46 (-0.27 - 1.23) |
|  | *B. valaisiana* | -0.06 (-0.64 - 0.51) | 0.52 (-0.25 - 1.31) |
| Life stage x Pheasant release | *B. garinii* |  | 0.81 (-0.23 - 1.88) |
|  | *B. valaisiana* | -0.63 (-1.63 - 0.32) | **1.45 (0.34 - 2.51)** |

**Supplementary references**

Abel, U., R. Schosser, and J. Süss. 1999. “Estimating the Prevalence of Infectious Agents Using Pooled Samples: Biometrical Considerations.” *Zbl Bakt*, **289** no, 5–7, 550-563.

[Scelza, B.A., S. P. Prall, N. Swinford, et al. 2020. “High Rate of Extrapair Paternity in a Human Population Demonstrates Diversity in Human Reproductive Strategies.” *Science advances* **6** no, 8: eaay6195.](http://paperpile.com/b/a5NgFR/D5FT)
